# Perioperative and Oncological Outcomes of Colorectal Cancer Surgery in Obese Patients: A Multicenter Retrospective Study

**DOI:** 10.1002/ags3.70156

**Published:** 2025-12-19

**Authors:** Kazuhiro Taguchi, Manabu Shimomura, Wataru Shimizu, Satoshi Ikeda, Masanori Yoshimitsu, Mohei Kouyama, Masahiro Nakahara, Hironori Kobayashi, Masatoshi Kochi, Yosuke Shimizu, Daisuke Sumitani, Shoichiro Mukai, Yuji Takakura, Yasuyo Ishizaki, Shinya Kodama, Masahiko Fujimori, Hiroshi Okuda, Takuya Yano, Tomoaki Bekki, Atsuhiro Watanabe, Saki Sato, Toshiyuki Moriuchi, Shohei Shiozaki, Kazuki Matsubara, Mizuki Yamaguchi, Tomohiro Adachi, Sho Ishikawa, Hideki Ohdan

**Affiliations:** ^1^ Department of Gastroenterological and Transplant Surgery, Graduate School of Biomedical and Health Sciences Hiroshima University Hiroshima Japan; ^2^ Department of Surgery Hiroshima City North Medical Center Asa Citizens Hospital Hiroshima Japan; ^3^ Department of Gastroenterological Surgery Hiroshima Prefectural Hospital Hiroshima Japan; ^4^ Department of Surgery Hiroshima City Hiroshima Citizens Hospital Hiroshima Japan; ^5^ Department of Surgery Hiroshima General Hospital Hatsukaichi Japan; ^6^ Department of Surgery Onomichi General Hospital Onomichi Japan; ^7^ Department of Surgery Hiroshima Memorial Hospital Hiroshima Japan; ^8^ Department of Gastroenterological Surgery National Hospital Organization Higashihiroshima Medical Center Higashihiroshima Japan; ^9^ Department of Surgery Kure Medical Center/Chugoku Cancer Center, Institute for Clinical Research Kure Japan; ^10^ Department of Surgery Hiroshima Prefectural Hospital Organization Futabanosato Prefectural Hospital Hiroshima Japan; ^11^ Department of Surgery Chugoku Rosai Hospital Kure Japan; ^12^ Department of Surgery Chuden Hospital Hiroshima Japan; ^13^ Department of Surgery Hiroshima‐Nishi Medical Center Hiroshima Japan; ^14^ Department of Surgery Yoshida General Hospital Akitakata Japan; ^15^ Department of Surgery Kure City Medical Association Hospital Kure Japan

**Keywords:** body mass index, colorectal surgery, lymph node dissection, obesity, surgical outcome, the log odds of positive lymph nodes

## Abstract

**Background:**

With global rising rates of obesity, surgeries for colorectal cancer (CRC) in obese patients are increasingly common. Although obesity may complicate surgical procedures and oncologically unbeneficial, its true clinical impact remains unclear.

**Methods:**

Patients with stage I–IV CRC who underwent surgical resection between 2017 and 2019 were enrolled from a prefecture‐wide multicenter database. Patients were categorized based on body mass index (BMI) into non‐obesity (BMI < 25 kg/m^2^), mild‐obesity (BMI ≥ 25 and < 30 kg/m^2^), and severe‐obesity (BMI ≥ 30 kg/m^2^) groups. Perioperative outcomes, long‐term survival, and lymph node metrics, including the log odds of positive lymph nodes (LODDS).

**Results:**

Among 2905 patients, 2283 were non‐obesity, 576 mild‐obesity, and 91 severe‐obesity. Younger, more often male, and had higher American Society of Anesthesiologists (ASA) class and Charlson Comorbidity Index scores are included in obesity group. Operative time and blood loss increased with BMI, while the number of retrieved lymph nodes and D3 dissection rates declined. Postoperative complications and hospital stay showed no significant differences. While recurrence‐free survival (RFS) and overall survival (OS) were comparable across BMI groups, obese patients had a higher proportion of LODDS ≥ −0.7. In stage III patients, LODDS ≥ −0.7 was more strongly associated with recurrence and poor prognosis in the obese subgroup.

**Conclusions:**

Obesity is associated with increased surgical complexity and limited pathological lymph node evaluation. Among stage III CRC patients, impaired nodal stratification—as reflected by elevated LODDS—may contribute to poorer prognosis in the obese population, highlighting the need for tailored oncological strategies.

## Graphical abstract

The prognostic value of the log odds of positive lymph nodes (LODDS) across BMI categories was investigated in patients undergoing colorectal cancer resection. The exploratory evidence demonstrates that LODDS serves as a strong prognostic marker, and its adverse effect may be more pronounced in patients with higher BMI.

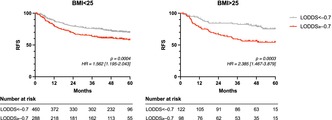

## 1 Introduction

The global rise in obesity has led to an increasing number of obese patients undergoing colorectal cancer (CRC) surgery [1]. Obesity has traditionally been considered a risk factor for adverse perioperative outcomes because of increased technical challenges in surgeries and higher rates of surgical complications [2, 3]. This trend is not limited to Western countries; obesity is also on the rise in Asian populations [4] underscoring the growing need for optimized surgical strategies tailored to obese patients in these regions [5].

Nevertheless, with the introduction of minimally invasive approaches such as laparoscopic surgery and parallel improvements in perioperative managements in recent years, some of these concerns have been lessened. As a results, the short‐term safety of CRC surgery in obese patients has improved substantially [5, 6, 7, 8, 9, 10].

In addition to perioperative outcomes, obesity has been suggested influence long‐term prognosis. Several studies have reported conflicting findings regarding the long‐term outcomes in obese CRC patients. While some data suggest that obesity may confer a survival advantage—the so‐called “obesity paradox”– [11, 12, 13] other report highlights compromised oncological quality in obese patients, such as inadequate lymph node dissection [14]. This limitation may hinder accurate cancer staging and thereby survival outcomes. The problem is especially in stage III disease, where thorough nodal evaluation guides decisions on adjuvant chemotherapy.

In clinical practice, even when the same extent of lymph nodes dissection is performed, the number of examined lymph nodes tends to be lower in obese patients [15], while obesity was not identified as a factor for lower examined lymph nodes in a meta‐analysis [16]. This may be explained by technical difficulty in pathological assessment with abundant adipose tissue, which could contribute to under detection of metastatic nodes. Actually, inadequate examination of lymph nodes is associated with poor prognosis after colorectal surgery [17, 18, 19, 20, 21].

One emerging metric to evaluating lymph node status is the log odds of positive lymph nodes (LODDS), which integrates both the number of positive and examined lymph nodes and may provide more robust prognostic information than conventional nodal staging systems [22, 23, 24]. However, the relevance of LODDS in the context of obesity remains unclear.

Therefore, in this multicenter retrospective study, we aimed to evaluate the short‐ and long‐term outcomes of CRC surgery in obese patients, with a particular attention on the quality of lymphadenectomy and the prognostic significance of LODDS. In addition, we explored whether obesity modifies the prognostic value of LODDS, especially in stage III CRC.

## 2 Methods

### 2.1 Study Population

We retrospectively reviewed data from 3164 consecutive patients who underwent colorectal resection for histologically confirmed or clinically suspected adenocarcinoma between January 2017 and December 2019 at 15 institutions affiliated with the Hiroshima Surgical Study Group of Clinical Oncology, a multicenter collaborative database. Patients were excluded if they had multiple primary cancers (*n* = 148), underwent simultaneous cholecystectomy (*n* = 73), received robotic‐assisted surgery (*n* = 30), or had missing data for height or weight (*n* = 8). Patient who had received neoadjuvant chemotherapy or radiotherapy before surgery were excluded from the cohort. A total of 2905 patients were included in the final analysis.

For each patient, we collected demographic and clinical information including age, sex, body mass index (BMI), American Society of Anesthesiologists (ASA) physical status classification, World Health Organization performance status (PS), Charlson Comorbidity Index (CCI), and comorbidities. Tumor‐related variables included tumor location, TNM classification, clinical tumor stage, and preoperative serum levels of carcinoembryonic antigen (CEA) and carbohydrate antigen 19‐9 (CA19‐9). Tumor staging was performed according to the 8th edition of UICC TNM Classification [25]. Surgical details such as operative approach (laparoscopic or open), and extent of lymph node dissection were recorded. Pathological findings included the total number of examined lymph nodes, number of metastatic lymph nodes, pathological tumor stage. Chemical fat‐clearing methods were not utilized for lymph node detection; however, comprehensive manual examination of mesenteric tissues was conducted to ensure thorough lymph node retrieval in accordance with standardized protocols in Japan.

Postoperative data included operative time, estimated blood loss, intraoperative blood transfusion, postoperative complications, mortality, and length of hospital stay. The patients were followed for up to 5 years, and oncological outcomes were evaluated in terms of recurrence‐free survival (RFS) and overall survival (OS). For stage III patients, information on adjuvant chemotherapy (ACT) administration (yes or no) was retrieved from the database and incorporated into the analyses. Additionally, nodal status was analyzed using the log odds of positive lymph nodes (LODDS), defined as the natural logarithm of the ratio of the number of metastatic lymph nodes plus 0.5 to the number of negative lymph nodes plus 0.5 (i.e., total examined lymph nodes minus metastatic nodes) plus 0.5. A detailed formula and justification are provided in the Supplementary Methods.

In accordance with Japan Society for Cancer of the Colon and Rectum (JSCCR) guidelines, D3 lymphadenectomy was considered indicated for tumors invading the muscularis propria or deeper (T2‐T4), as well as for all clinically node‐positive cases. Lower rectal (Rb) tumors were assessed according to proximal lymph node dissection criteria; information on lateral lymph node dissection was insufficient due to lack of data in the database.

Because only patients who underwent resection of the primary tumor were included in this study, the surgical intent for stage IV cases was primary tumor resection. Although surgical intent may influence perioperative management and lymphadenectomy, detailed information on curability (R0–R3) was not available in the database.

The study was approved by the institutional review board at each participating institution.

### 2.2 Body Mass Index (BMI) Classification

Patients were classified into three groups based on BMI, according to the World Health Organization (WHO) criteria for Asian populations. Specifically, individuals with a BMI of less than 25 kg/m^2^ were defined as non‐obesity, those with a BMI between 25.0 and 29.9 kg/m^2^ were considered mild‐obesity, and those with a BMI of 30.0 kg/m^2^ or greater were classified as severe‐obesity. This stratification allowed for a systematic comparison of perioperative and oncological outcomes across different degrees of obesity in the study population.

In accordance with the Asia‐Pacific BMI classification, which defines obesity as BMI ≥ 25 kg/m^2^ in East Asian populations, patients in this study with BMI ≥ 25 kg/m^2^ were categorized as obese. This terminology was used consistently throughout the manuscript. For the primary analyses, BMI was categorized into three groups (< 25, 25–29.9, and ≥ 30). Because the number of patients with BMI ≥ 30 was relatively small in this cohort, BMI was dichotomized as < 25 versus ≥ 25 in exploratory subgroup survival analyses to improve interpretability and analytic stability.

### 2.3 Statistical Analysis

Statistical analyses were performed to compare clinical, perioperative, and oncological variables among the three BMI groups (non‐obesity, mild‐obesity, and severe‐obesity). Categorical variables were compared using Pearson's chi‐squared test or Fisher's exact test, as appropriate. Continuous variables were expressed as medians with ranges, and were analyzed using one‐way analysis of variance (ANOVA) for normally distributed data, the Kruskal–Wallis test for non‐normally distributed data.

Survival outcomes, including recurrence‐free survival (RFS) and OS, were estimated using the Kaplan–Meier method. Differences between survival curves were evaluated using the log‐rank test. Hazard ratios (HRs) and corresponding 95% confidence intervals (CIs) were calculated using Cox proportional hazards regression models to identify prognostic factors associated with recurrence and mortality.

All statistical analyses were performed using GraphPad Prism version 10.6.0 (GraphPad Software, San Diego, CA, USA) and JMP Student Edition 18.2.1 (SAS Institute Inc., Cary, NC, USA). A two‐sided *p*‐value of < 0.05 was considered statistically significant.

## 3 Results

### 3.1 Patient and Oncological Characteristics

**FIGURE 1:**
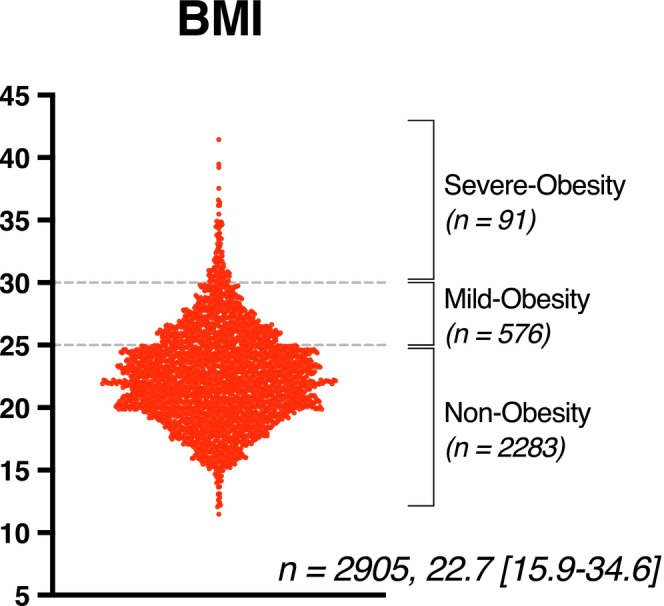
Distribution of body mass index (BMI) in the study cohort. The violin plot illustrates the distribution of BMI among all 2905 patients included in the study. Patients were stratified into three groups based on BMI: Non‐obesity (BMI < 25, *n* = 2283), mild‐obesity (25 ≤ BMI < 30, *n* = 576), and severe‐obesity (BMI ≥ 30, *n* = 91). The median BMI was 22.7 (range: 15.9–34.6).

A total of 2905 patients were included in the analysis, consisting of 2238 non‐obese patients (BMI < 25), 576 mildly obese patients (25 ≤ BMI < 30), and 91 severely obese patients (BMI ≥ 30) (Figure 1). Obese patients were significantly younger (median age: non‐obese 72, mildly obese 71, severely obese 69; *p* = 0.0001), and more likely to be male (*p* = 0.0033). ASA class scores, CCI values, and tumor location varied significantly among BMI groups (*p* < 0.0001, *p* = 0.0416, and *p* = 0.0181, respectively) (Table 1). Differences were also noted in *T* classification (*p* = 0.0399), although *N* and *M* classifications showed no significant differences. There were no significant differences in clinical stage distribution among the BMI groups, nor in baseline tumor marker levels.

**TABLE 1:** Baseline demographic and tumor characteristics stratified by BMI category.

	Non‐obesity BMI < 25 *n* = 2238	Mild‐obesity BMI ≥ 25, < 30 *n* = 576	Severe‐obesity BMI ≥ 30 *n* = 91	*p*
**Sex**				0.0033
Male	1140 (50.9%)	338 (58.7%)	51 (56.0%)	
Female	1098 (49.1%)	238 (41.3%)	40 (44.0%)	
Age (year old) [median (range)]	72 (23–102)	71 (22–91)	69 (42–89)	0.0001
BMI [median (range)]	21.3 (11.4–24.9)	26.6 (25.0–29.9)	31.4 (30.0–41.5)	< 0.0001
**ASA class**				< 0.0001
1	451 (20.2%)	71 (12.4%)	6 (6.7%)	
2	1473 (66.1%)	420 (73.3%)	64 (71.1%)	
3	301 (13.5%)	81 (14.1%)	19 (21.1%)	
4	5 (0.2%)	1 (0.2%)	1 (1.1%)	
**Performance status**				0.1116
0	1511 (67.5%)	403 (70.1%)	66 (72.5%)	
1	471 (21.1%)	131 (22.8%)	15 (16.5%)	
2	177 (7.9%)	30 (5.2%)	8 (8.8%)	
3	60 (2.7%)	8 (1.4%)	2 (2.2%)	
4	18 (0.8%)	3 (0.5%)	0 (0%)	
**Charlson comorbidity index**				0.0416
0	1271 (56.8%)	302 (52.4%)	39 (42.9%)	
1	485 (21.7%)	135 (23.4%)	22 (24.2%)	
2	260 (11.6%)	85 (14.8%)	17 (18.7%)	
≥ 3	222 (9.9%)	54 (9.4%)	13 (14.2%)	
**Tumor location** *				0.0181
C	222 (9.9%)	55 (9.6%)	11 (12.1%)	
A	434 (19.4%)	102 (17.7%)	17 (18.7%)	
T	241 (10.8%)	45 (7.8%)	3 (3.3%)	
D	118 (5.3%)	31 (5.4%)	6 (6.6%)	
S	525 (23.5%)	170 (29.5%)	29 (31.9%)	
RS	296 (13.2%)	66 (11.5%)	15 (16.5%)	
Ra, Rb, P	402 (18.0%)	107 (18.6%)	10 (11.0%)	
***T* factor**				0.0399
Tis	10 (0.5%)	5 (0.9%)	2 (2.2%)	
T1a	26 (1.2%)	6 (1.0%)	1 (1.1%)	
T1b	413 (18.4%)	130 (22.6%)	13 (14.3%)	
T2 T3	339 (15.2%) 831 (37.1%)	98 (17.0%) 215 (37.3%)	16 (17.6%) 36 (39.6%)	
T4a	508 (22.7%)	105 (18.2%)	20 (22.0%)	
T4b	110 (4.9%)	15 (2.6%)	3 (3.3%)	
***N* factor**				0.1777
N0	1602 (71.6%)	431 (74.8%)	64 (70.3%)	
N1	461 (20.6%)	105 (18.2%)	22 (24.2%)	
N2	129 (5.7%)	32 (5.6%)	4 (4.4%)	
N3	46 (2.1%)	8 (1.4%)	1 (1.1%)	
***M* factor**				0.6792
M0	2211 (98.8%)	572 (99.3%)	89 (97.8%)	
M1a	19 (0.9%)	4 (0.7%)	2 (2.2%)	
M1b	4 (0.2%)	0 (0%)	0 (0%)	
M1c1	1 (0.04%)	0 (0%)	0 (0%)	
M1c2	3 (0.1%)	0 (0%)	0 (0%)	
**Clinical stage**				0.1171
0	10 (0.4%)	5 (0.9%)	2 (2.2%)	
I	751 (33.6%)	221 (38.8%)	28 (30.8%)	
IIa	567 (25.3%)	147 (25.5%)	24 (26.4%)	
IIb	229 (10.2%)	50 (8.7%)	8 (8.8%)	
IIc	36 (1.6%)	5 (0.9%)	2 (2.2%)	
IIIa	21 (0.9%)	12 (2.1%)	2 (2.2%)	
IIIb	439 (19.6%)	97 (16.8%)	20 (22.0%)	
IIIc	157 (7.0%)	33 (5.7%)	3 (3.3%)	
IVa	19 (0.9%)	4 (0.7%)	2 (2.2%)	
IVb	4 (0.2%)	0 (0%)	0 (0%)	
IVc	4 (0.2%)	0 (0%)	0 (0%)	
CEA (ng/mL) [median (range)]	3.2 (0–974.1)	3.1 (0–325.0)	3.8 (0–152.3)	0.7479
CA19‐9 (U/mL) [median (range)]	9.4 (0–4623.1)	8.2 (0–8716.4)	8.3 (0–125.6)	0.7069

* Tumor locations: C, cecum; A, ascending colon; T, transverse colon; D, descending colon; S, sigmoid colon; RS, rectosigmoid; Ra, Rb, P, rectum.

Abbrevations: ASA, American society of anesthesiologists; BMI, body mass index; CA19‐9, carbohydrate antigen 19–9; CEA, carcinoembryonic antigen.

### 3.2 Surgical Procedures and Perioperative Outcomes

**FIGURE 2:**
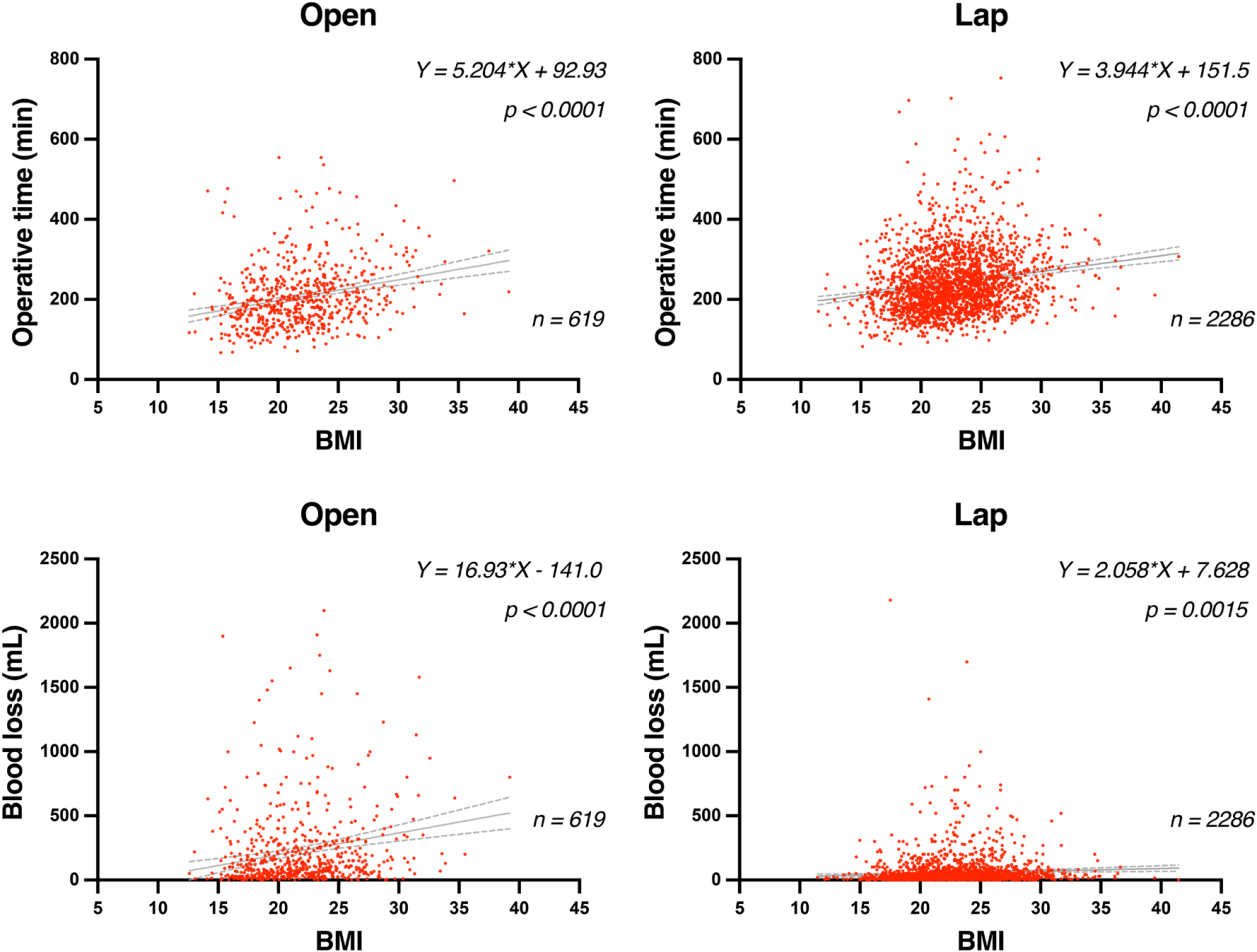
Association between body mass index (BMI) and intraoperative parameters stratified by surgical approach. (Top panels) Scatter plots show the relationship between BMI and operative time (minute) in patients undergoing open surgery (left, *n* = 619) and laparoscopic surgery (right, *n* = 2286). (Bottom panels) Scatter plots illustrate the relationship between BMI and estimated intraoperative blood loss (mL) in patients undergoing open surgery (left, *n* = 619) and laparoscopic surgery (right, *n* = 2286). Linear regression lines with 95% confidence intervals (dotted lines) are shown. Regression equations and *p*‐values are indicated in each panel. BMI, body mass index; Lap, laparoscopic.

The laparoscopic approach was more frequently used across all BMI categories, although the rate of open surgery was slightly higher in the severely obese group (24.2%) compared to the non‐obese group (22.2%) (*p* = 0.0284). The extent of lymph node dissection differed significantly by BMI group (*p* = 0.0163), with a decreasing trend in D3 dissections observed with increasing BMI. The number of examined lymph nodes was significantly lower in the obese groups (median: non‐obese 19, mild‐obesity 17, severe‐obesity 15; *p* < 0.0001), while the number of metastatic nodes was not significantly different (*p* = 0.8233). Operative time (*p* < 0.0001) and blood loss (*p* < 0.0001) increased significantly with higher BMI (Table 2). Scatter plots demonstrated significant positive correlations between BMI and operative time, both in open (*p* < 0.0001) and laparoscopic surgery (*p* < 0.0001). Similarly, blood loss was positively associated with BMI in both open (*p* < 0.0001) and laparoscopic approaches (*p* = 0.0015), with a more pronounced effect seen in open surgery (Figure 2). The proportion of patients undergoing D3 lymphadenectomy decreased with increasing BMI, particularly in the open approach group (*p* = 0.0418), with only 40.9% of severely obese patients receiving D3 dissection compared to 66.8% of non‐obese patients. A similar trend was noted in patients with clear indication for D3 dissection (*p* = 0.0197), although no significant difference was observed in the laparoscopic subgroup (*p* = 0.4269) (Table 3).

**TABLE 2:** Surgical characteristics and short‐term outcomes of patients according to BMI category.

	Non‐obesity BMI < 25 *n* = 2238	Mild‐obesity BMI ≥ 25, < 30 *n* = 576	Severe‐obesity BMI ≥ 30 *n* = 91	*p*
**Surgical approach**				0.0284
Open	497 (22.2%)	100 (17.4%)	22 (24.2%)	
Laparoscopic	1741 (77.8%)	476 (82.6%)	69 (75.8%)	
**LN dissection**				0.0163
D0	7 (0.3%)	0 (0%)	1 (1.1%)	
D1	49 (2.2%)	5 (0.9%)	4 (4.4%)	
D2	664 (29.7%)	193 (33.5%)	32 (35.2%)	
D3	1518 (67.8%)	378 (65.6%)	54 (59.3%)	
# of examined LN [median (range)]	19 (0–116)	17 (0–84)	15 (0–63)	< 0.0001
# of pathological metastatic LN [median (range)]	0 (0–24)	0 (0–25)	0 (0–15)	0.8233
Operative time (min) [median (range)]	213 (67–702)	237 (98–753)	280 (140–497)	< 0.0001
Blood loss (mL) [median (range)]	30 (0–2181)	40 (0–1450)	50 (0–5100)	< 0.0001
**Intraoperative blood transfusion**				0.2440
Yes	73 (3.3%)	12 (2.1%)	4 (4.9%)	
No	2161 (96.7%)	561 (97.9%)	87 (95.6%)	
**Postoperative complication**				0.7305
Yes	600 (26.8%)	149 (25.9%)	27 (29.7%)	
No	1635 (73.2%)	427 (74.1%)	64 (70.3%)	
Postoperative length of hospital stay (day) [median (range)]	11 (1–389)	10 (5–388)	11 (6–64)	0.2319

Abbreviations: BMI, body mass index; LN, lymph node; min, minute; mL, milliliter.

**TABLE 3:** Comparison of the extent of lymph node dissection (D ≤ 2 or D3) stratified by surgical approach and BMI category.

	Non‐obesity BMI < 25	Mild‐obesity BMI ≥ 25, < 30	Severe‐obesity BMI ≥ 30	*p*
**All (*n* = 2905)**				
*Open (n = 619)*				0.0418
≤ D2	165 (33.2%)	36 (36.0%)	13 (59.1%)	
D3	332 (66.8%)	64 (64.0%)	9 (40.9%)	
*Laparoscopic (n = 2286)*				0.6161
≤ D2	555 (31.9%)	162 (34.0%)	24 (34.8%)	
D3	1186 (68.1%)	314 (66.0%)	45 (65.2%)	
**Indication of D3 Dissection (*n* = 2323)**				
*Open (n = 590)*				0.0197
≤ D2	145 (30.6%)	33 (34.4%)	12 (60.0%)	
D3	329 (69.4%)	63 (65.6%)	8 (40.0%)	
*Laparoscopic (n = 1733)*				0.4269
≤ D2	267 (20.0%)	65 (18.8%)	15 (26.3%)	
D3	1064 (80.0%)	280 (81.2%)	42 (73.7%)	

Abbrevations: BMI, body mass index; D ≤ 2, dissection up to D2 level; D3, level 3 lymph node dissection (according to Japanese classification of Colorectal, appendiceal, and anal carcinoma).

### 3.3 Survival Outcomes by BMI Category

Kaplan–Meier analysis revealed no significant differences in recurrence‐free survival (RFS) or OS among BMI categories for Stage I, II, or III colorectal cancer. Five‐year RFS and OS curves were largely overlapping across BMI groups (Figure S1).

### 3.4 Association Between BMI and Lymph Node Metrics

**FIGURE 3:**
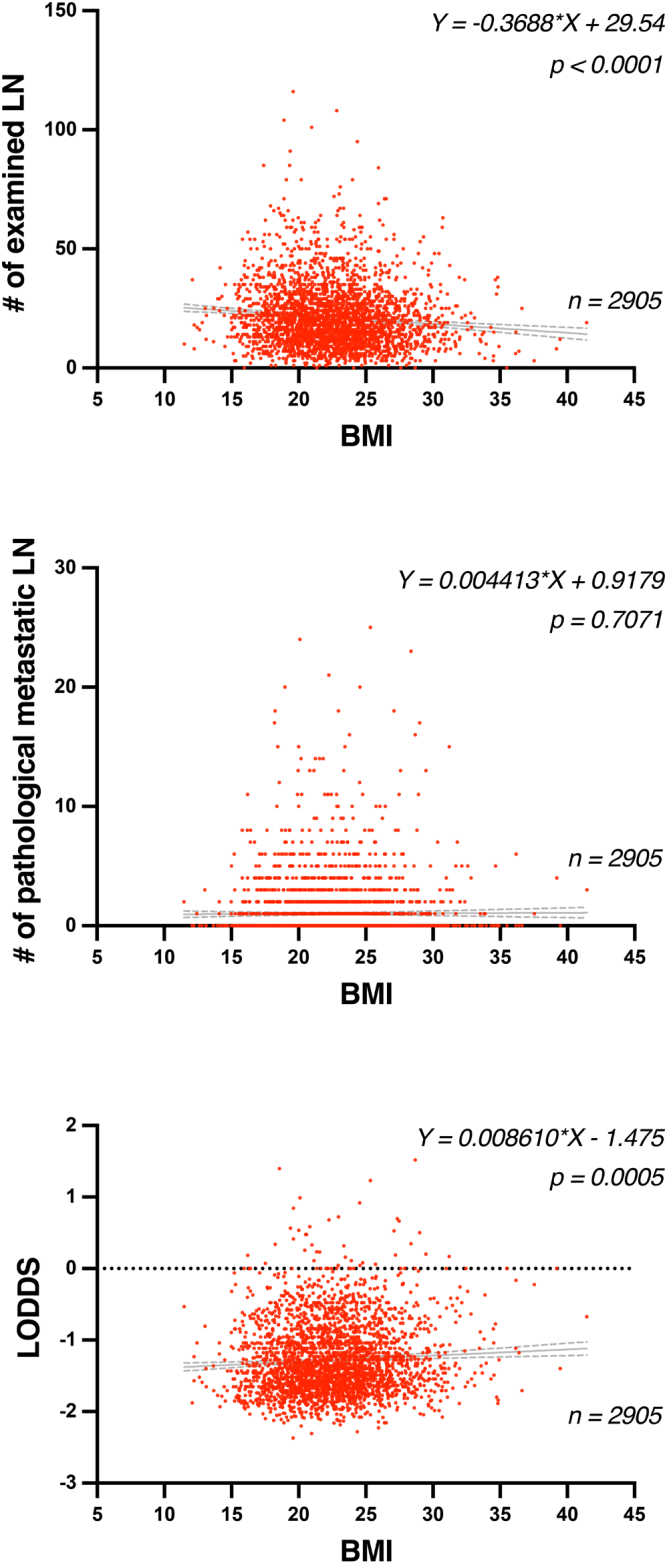
Association between body mass index (BMI) and lymph node parameters. Scatter plots illustrating the associations between BMI and (top) the number of examined lymph nodes, (middle) the number of pathological metastatic lymph nodes, and (bottom) the log odds of positive lymph nodes (LODDS) in all patients (*n* = 2905). Linear regression lines with 95% confidence intervals (dashed lines) are shown. Each dot represents an individual case. Regression equations and *p*‐values are indicated on each panel. BMI, body mass index; LN, lymph node; LODDS, log odds of positive lymph nodes.

Higher BMI was significantly associated with a reduced number of examined lymph nodes (*p* < 0.0001), while no correlation was observed with the number of metastatic nodes. Interestingly, LODDS values increased significantly with BMI (*p* = 0.0005), indicating a trend toward unfavorable nodal status in obese patients (Figure 3). According to the guideline for appropriate evaluation of status of metastatic lymph nodes, more than 12 examined lymph nodes are recommended. A significantly higher proportion of obese patients had < 12 examined lymph nodes (non‐obese 21.4%, severe obesity 28.6%; *p* = 0.0051) (Table S1). Furthermore, we focused on LODDS which not only reflects nodal metastasis but is also influenced by the total number of examined lymph nodes, and thus may serve as a surrogate marker for staging adequacy and recurrence risk. An ROC analysis identified −0.7 as optimal LODDS cut‐off for predicting recurrence. The ROC curve yielded an AUC of 0.6699, with a sensitivity of 0.7467 and specificity of 0.4333 for predicting recurrence (Figure S2). The proportion of patients with LODDS ≥ −0.7, considered a poor prognostic factor, increased with BMI (non‐obese 13.8%, severe‐obesity 24.2%; *p* = 0.0366) (Table S1).

### 3.5 Prognostic Significance of LODDS According to BMI in Stage III CRC

Among the 969 patients with pathological Stage III colorectal cancer, stratification by LODDS and BMI revealed distinct prognostic implications. Based on ROC analysis, a LODDS cut‐off value of −0.7 was determined to optimally discriminate recurrence risk.

In the non‐obese group (BMI < 25, *n* = 749), patients with LODDS ≥ –0.7 had a significantly higher recurrence rate compared to those with LODDS < –0.7 (30.9% vs. 21.0%, *p =* 0.0026). Similarly, in the obese group (BMI ≥ 25, *n* = 220), recurrence was significantly more frequent in LODDS ≥ –0.7 group (34.7% vs. 17.2%, *p =* 0.0029) (Table 4).

**TABLE 4:** Association between LODDS and recurrence according to BMI in patients with Stage III colorectal cancer.

	LODDS < −0.7	LODDS ≥ −0.7	*p*
**Stage III (*n* = 969)**			
*BMI < 25 (n = 749)*			0.0026
Recurrence (−)	364 (79.0%)	199 (69.1%)	
Recurrence (+)	97 (21.0%)	89 (30.9%)	
*BMI ≥ 25 (n = 220)*			0.0029
Recurrence (−)	101 (82.8%)	64 (65.3%)	
Recurrence (+)	21 (17.2%)	34 (34.7%)	

Abbrevations: BMI, body mass index; LN, lymph node; LODDS, log odds of positive lymph nodes.

**FIGURE 4:**
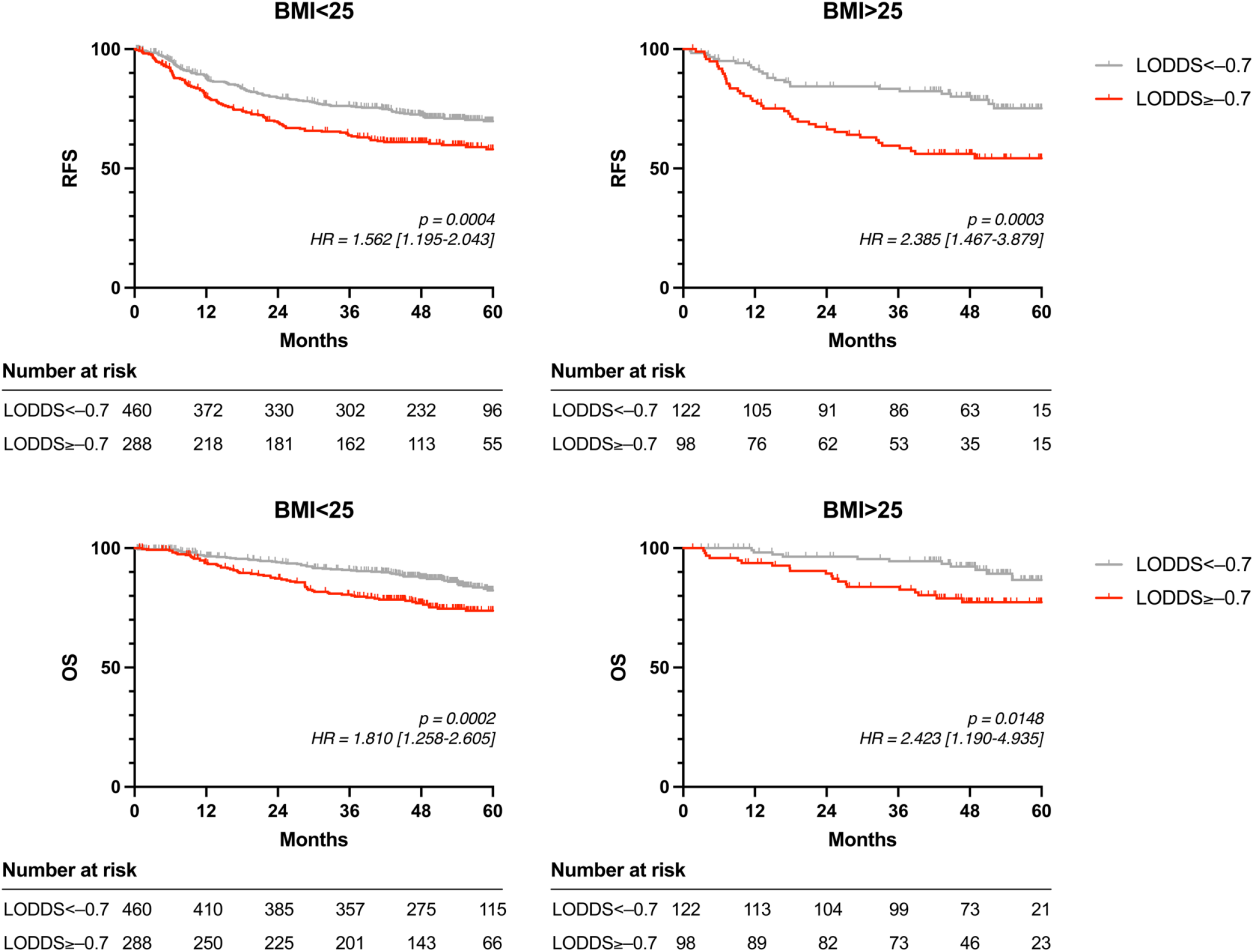
Prognostic impact of LODDS stratified by BMI category in Stage III colorectal cancer. Kaplan–Meier curves of recurrence‐free survival (RFS, top panels) and overall survival (OS, bottom panels) in patients with Stage III colorectal cancer stratified by BMI groups: BMI < 25 (left) and BMI ≥ 25 (right). Patients were further categorized based on LODDS values (< −0.7 vs. ≥ −0.7). Survival differences were evaluated using the log‐rank test, and hazard ratios (HR) and corresponding 95% confidence intervals (CIs) (bracket) were calculated using Cox proportional hazards model. The number of patients at risk is displayed below each curve. LODDS, log odds of positive lymph nodes.

In exploratory subgroup analyses, BMI was dichotomized as < 25 versus ≥ 25 due to the limited number of patients with BMI ≥ 30 and to enhance the interpretability of survival curves. Kaplan–Meier analysis confirmed these trends. Among non‐obese patients, LODDS ≥ –0.7 was significantly associated with worse RFS (*p =* 0.0004, HR = 1.562) and OS (*p =* 0.0002, HR = 1.810). In the obese group, the prognostic impact of LODDS ≥ –0.7 was even more pronounced (RFS: *p =* 0.0003, HR = 2.385; OS: *p =* 0.0148, HR = 2.423) (Figure 4). In subgroup analysis, the association between high LODDS and poor survival appeared larger in patients with BMI ≥ 25; however, the LODDS × BMI interaction term was not statistically significant.

Given the potential impact of ACT on survival outcomes, we evaluated ACT utilization across BMI and LODDS categories. ACT utilization did not significantly differ across BMI or LODDS categories in stage III patients (Table S2). To further assess the prognostic impact of LODDS independent of ACT, we generated RFS and OS curves stratified by ACT status within each BMI group (Figure S3). Among patients with BMI < 25, those with LODDS ≥ –0.7 showed significantly worse survival in the ACT(−) subgroup, whereas in patients with BMI ≥ 25, high LODDS remained a strong adverse prognostic factor even among those who received ACT.

These findings underscore the value of LODDS as a robust, prognostic indicator in Stage III colorectal cancer. The association between high LODDS and poor survival appeared larger in patients with higher BMI, although this should be interpreted as an exploratory observation.

## 4 Discussion

In this large‐scale multicenter cohort study of over 2900 patients undergoing curative‐intent colorectal cancer surgery, we demonstrated that elevated BMI was associated with increased perioperative difficulty, reduced lymph node yield, and higher LODDS values—despite comparable oncologic staging and baseline tumor burden. Notably, while BMI itself was not directly associated with recurrence or survival, the LODDS metric emerged as a powerful prognostic marker, particularly among obese patients with Stage III disease. These findings highlight the complex interplay between obesity, surgical quality metrics, and oncologic outcomes.

Obesity is known to pose technical challenges in abdominal surgery, and our results support these concerns. As BMI increased, operative time and blood loss rose significantly, and D3 lymphadenectomy rates declined—particularly in open surgery. These findings suggest that elevated BMI may compromise surgical thoroughness, particularly in nodal dissection, likely because of increased visceral fat and limited operative field. Notably, this trend was especially pronounced in open surgery and persisted even in patients with clear indications for D3 lymphadenectomy. Interestingly, laparoscopic surgery appeared to mitigate this decline, maintaining comparable D3 rates across BMI groups. However, this technical advantage did not translate into improved long‐term outcomes, suggesting that minimally invasive approaches alone may be insufficient to overcome the oncological challenges posed by obesity. These observations raise concerns about surgical equity in the obese population.

Accurate nodal evaluation is essential for staging and guiding adjuvant therapy in colorectal cancer. Our study revealed that a higher BMI correlated with fewer examined lymph nodes, with nearly 30% of severely obese patients failing to meet the benchmark of ≥ 12 nodes. This deficit in nodal yield could result in staging underestimation and inappropriate treatment stratification. In obese patients, inadequate lymph node assessment may arise not only from surgical limitations in the extent of dissection but also from technical challenges during pathological examination. Even when nodal tissues are surgically resected in an oncologically adequate manner, abundant mesenteric fat in obese individuals can obscure small lymph nodes, leading to their omission during pathological sampling and counting. As a result, the number of positive lymph nodes may be underrepresented, giving the impression a lower nodal stage. This raises the concern that patients who would otherwise qualify for ACT—particularly those with Stage III disease—may not receive appropriate postoperative treatment due to this staging bias. LODDS, a metric that adjusts for both positive and negative lymph nodes, was significantly elevated in obese patients, likely reflecting both compromised nodal clearance and tumor burden. Unlike absolute node counts, LODDS offers a more nuanced assessment and may better capture the prognostic significance of nodal status in technically challenging surgeries.

Our study is, to our knowledge, the first to examine how the prognostic significance of LODDS varies across BMI subgroups in Stage III colorectal cancer. Among patients with LODDS ≥ –0.7, recurrence rates were higher, and this association appeared more pronounced in patients with higher BMI. This suggests that LODDS not only serves as a surrogate for nodal burden but may also reflect the adequacy of surgical resection. Furthermore, the interaction between BMI and LODDS implies that traditional nodal metrics may be insufficient to stratify risk in obese populations, underscoring the need for tailored surgical benchmarks and follow‐up strategies. Although the adverse prognostic impact of high LODDS appeared more pronounced in patients with higher BMI, the LODDS × BMI interaction was not statistically significant. Therefore, this observation is exploratory and should be interpreted with caution. In patients with BMI ≥ 25, high LODDS remained a strong adverse prognostic factor even among those who received ACT. Although exploratory, this finding might suggest that in obese patients, high LODDS reflect more aggressive tumor biology that is not fully mitigated by standard ACT.

Given the rising global prevalence of obesity, our findings carry significant implications. First, surgeons should be cognizant of potential under‐dissection in obese patients and consider additional training, intraoperative strategies, or use of minimally invasive techniques where feasible. Second, LODDS should be considered in postoperative risk stratification, especially in patients with high BMI. Future prospective studies should explore whether preoperative interventions (e.g., weight optimization) or intraoperative adjuncts (e.g., ICG guidance, robotic assistance) could improve nodal clearance and outcomes in obese patients.

This study has several limitations. First, its retrospective design may introduce selection bias, despite the large sample size. Second, detailed information regarding surgeon experience, surgical instrumentation, and visceral fat area was not available, all of which may influence the extent and accuracy of lymph node retrieval. Third, although LODDS demonstrated strong prognostic value, it remains underutilized in current clinical practice and warrants further validation in external cohorts before routine adoption. Because the ROC‐derived cut‐off was generated from the dataset, reproducibility could not be also evaluated, and the lack external validation represents an important limitation. Future studies using independent cohorts are required to confirm the generalizability of this cut‐off. Fourth, it is important to note that this study was conducted exclusively in a Japanese population. Given the known ethnic and regional differences in BMI distribution and body composition, the generalizability of our findings to Western populations or other ethnic groups may be limited. The definition and clinical significance of obesity remarkably differ from those between Asian populations and Western countries. Future multinational studies are warranted to validate the prognostic value of LODDS across diverse populations and to establish globally applicable surgical benchmarks for obese patients with colorectal cancer. Fifth, because detailed information on curability status (R0–R3) was not available in the database, we were unable to identify cases that underwent confirmed R0 resection. Therefore, an analysis restricted to R0 resections could not be performed. This limitation should be considered when interpreting the prognostic implications of lymph node metrics in this cohort. Lastly, our analysis did not reveal a direct difference in long‐term outcomes between obese and non‐obese groups, although LODDS remained a significant prognostic factor, particularly in obese patients. In addition, because our analyses were based on univariate comparisons without multivariate Cox adjustment, we cannot conclude that LODDS is an independent prognostic factor. The absence of multivariable modeling represents an important limitation. Therefore, it could not be concluded that obesity itself is an independent adverse prognostic factor. Indeed, despite the association between high BMI and elevated LODDS values, high BMI alone was not linked to poorer survival in our cohort. Future prospective studies with stratified subgroup analyses and mechanistic investigations are needed to clarify whether obesity independently contributes to adverse oncologic outcomes.

In summary, obesity was associated with inferior nodal evaluation and elevated LODDS in colorectal cancer surgery. While BMI alone did not predict survival, LODDS emerged as a robust prognostic marker—particularly among obese patients with Stage III disease. Our findings support the adoption of LODDS as a complementary risk stratification tool and underscore the need for heightened surgical vigilance in the obese population.

## 5 Conclusion

Obesity is associated with increased surgical complexity and reduced lymph node evaluation. Among patients with stage III colorectal cancer, impaired nodal stratification—as reflected by elevated LODDS—may contribute to poorer oncologic outcomes in the obese population. These findings underscore the need for tailored surgical and postoperative strategies to optimize oncological care in this growing patient group.

## Author Contributions

**Kazuhiro Taguchi:** conceptualization, methodology, software, data curation, supervision, formal analysis, validation, investigation, funding acquisition, visualization, project administration, resources, writing – review and editing, writing – original draft. **Manabu Shimomura:** conceptualization, data curation, methodology, funding acquisition, supervision, investigation, validation, project administration. **Wataru Shimizu:** data curation, investigation, validation. **Satoshi Ikeda:** data curation, validation, investigation. **Masanori Yoshimitsu:** data curation, investigation, validation. **Mohei Kouyama:** data curation, investigation, validation. **Masahiro Nakahara:** data curation, investigation, validation. **Hironori Kobayashi:** data curation, investigation, validation. **Masatoshi Kochi:** data curation, investigation, validation. **Yosuke Shimizu:** data curation, investigation, validation. **Daisuke Sumitani:** data curation, investigation, validation. **Shoichiro Mukai:** data curation, investigation, validation. **Yuji Takakura:** data curation, investigation, validation. **Yasuyo Ishizaki:** data curation, investigation, validation. **Shinya Kodama:** data curation, investigation, validation. **Masahiko Fujimori:** data curation, investigation, validation. **Hiroshi Okuda:** data curation, investigation, validation, project administration. **Takuya Yano:** data curation, investigation, validation, project administration. **Tomoaki Bekki:** data curation, investigation, validation. **Atsuhiro Watanabe:** data curation, investigation, validation. **Saki Sato:** data curation, investigation, validation. **Toshiyuki Moriuchi:** data curation, investigation, validation. **Shohei Shiozaki:** data curation, investigation, validation. **Kazuki Matsubara:** data curation, investigation, validation. **Mizuki Yamaguchi:** data curation, investigation, validation. **Tomohiro Adachi:** data curation, investigation, validation, formal analysis, software. **Sho Ishikawa:** software, data curation, investigation, validation, formal analysis. **Hideki Ohdan:** conceptualization, methodology, supervision, funding acquisition, project administration.

## Funding

The authors have nothing to report.

## Ethics Statement

Administrative permissions were not required to access and use the medical records described in this study. This study was authorized in advance by the institutional review board of the Hiroshima University Hospital (approval number: E2021‐2527).

## Consent

There is no need for consent to participate to be obtained due to retrospective study.

## Conflicts of Interest

Hideki Ohdan is an editor of Annals of Gastroenterological Surgery. The other authors declare no conflicts of interest.

## Supporting information


**Figure S1:** Kaplan–Meier survival curves for relapse‐free survival (RFS) and overall survival (OS) according to BMI category in stage I–III colorectal cancer patients.
**Figure S2:** ROC curve used to determine the optimal LODDS cut‐off for predicting recurrence.
**Figure S3:** Recurrence‐free survival (RFS) and overall survival (OS) according to LODDS and adjuvant chemotherapy (ACT) status in stage III colorectal cancer, stratified by BMI category.
**Table S1:** Association between BMI and Lymph Node Evaluation Parameters.
**Table S2:** Association between LODDS and adjuvant chemotherapy according to BMI in patients with Stage III colorectal cancer.

## References

[1] R. L. Siegel , N. S. Wagle , A. Cercek , R. A. Smith , and A. Jemal , “Colorectal Cancer Statistics, 2023,” CA: A Cancer Journal for Clinicians 73, no. 3 (2023): 233–254. 36856579 10.3322/caac.21772

[2] T. Kazi , T. McKechnie , Y. Lee , et al., “The Impact of Obesity on Postoperative Outcomes Following Surgery for Colorectal Cancer: Analysis of the National Inpatient Sample 2015–2019,” ANZ Journal of Surgery 94, no. 7–8 (2024): 1305–1312. 38888262 10.1111/ans.19135

[3] G. Bocca , S. Mastoridis , T. Yeung , D. R. C. James , and C. Cunningham , “Visceral‐To‐Subcutaneous Fat Ratio Exhibits Strongest Association With Early Post‐Operative Outcomes in Patients Undergoing Surgery for Advanced Rectal Cancer,” International Journal of Colorectal Disease 37, no. 8 (2022): 1893–1900. 35902393 10.1007/s00384-022-04221-8 PMC9388433

[4] M. Ng , T. Fleming , M. Robinson , et al., “Global, Regional, and National Prevalence of Overweight and Obesity in Children and Adults During 1980–2013: A Systematic Analysis for the Global Burden of Disease Study 2013,” Lancet 384, no. 9945 (2014): 766–781. 24880830 10.1016/S0140-6736(14)60460-8 PMC4624264

[5] M. Yamashita , T. Tominaga , T. Nonaka , et al., “Impact of Obesity on Short‐Term Outcomes of Laparoscopic Colorectal Surgery for Japanese Patients With Colorectal Cancer: A Multicenter Study,” Asian Journal of Endoscopic Surgery 14, no. 3 (2021): 432–442. 33111467 10.1111/ases.12888

[6] M. Camilleri , H. Malhi , and A. Acosta , “Gastrointestinal Complications of Obesity,” Gastroenterology 152, no. 7 (2017): 1656–1670. 28192107 10.1053/j.gastro.2016.12.052 PMC5609829

[7] Y. Fujita , K. Hida , N. Hoshino , et al., “Laparoscopic vs. Open Surgery for Rectal Cancer in Patients With Obesity: Short‐Term Outcomes and Relapse‐Free Survival Across Age Groups,” Surgery Today 55, no. 1 (2025): 10–17. 39102009 10.1007/s00595-024-02901-2

[8] A. Vignali , P. D. Nardi , L. Ghirardelli , S. D. Palo , and C. Staudacher , “Short and Long‐Term Outcomes of Laparoscopic Colectomy in Obese Patients,” World Journal of Gastroenterology 19, no. 42 (2013): 7405–7411. 24259971 10.3748/wjg.v19.i42.7405 PMC3831222

[9] T. Akiyoshi , M. Ueno , Y. Fukunaga , et al., “Effect of Body Mass Index on Short‐Term Outcomes of Patients Undergoing Laparoscopic Resection for Colorectal Cancer: A Single Institution Experience in Japan,” Surgical Laparoscopy, Endoscopy & Percutaneous Techniques 21, no. 6 (2011): 409–414. 10.1097/SLE.0b013e31822e5fdc 22146162

[10] K. Kazama , M. Numata , T. Aoyama , et al., “Laparoscopic vs. Open Surgery for Stage II/III Colon Cancer Patients With Body Mass Index > 25 Kg/m^2^ ,” In Vivo 34, no. 4 (2020): 2079–2085. 32606186 10.21873/invivo.12011 PMC7439910

[11] B. J. Caan , J. A. Meyerhardt , C. H. Kroenke , et al., “Explaining the Obesity Paradox: The Association Between Body Composition and Colorectal Cancer Survival (C‐SCANS Study),” Cancer Epidemiology, Biomarkers & Prevention 26, no. 7 (2017): 1008–1015. 10.1158/1055-9965.EPI-17-0200 PMC5647152 28506965

[12] M. C. Gonzalez , C. A. Pastore , S. P. Orlandi , and S. B. Heymsfield , “Obesity Paradox in Cancer: New Insights Provided by Body Composition 1, 2, 3,” American Journal of Clinical Nutrition 99, no. 5 (2014): 999–1005. 24572565 10.3945/ajcn.113.071399

[13] H. Lennon , M. Sperrin , E. Badrick , and A. G. Renehan , “The Obesity Paradox in Cancer: A Review,” Current Oncology Reports 18, no. 9 (2016): 56. 27475805 10.1007/s11912-016-0539-4 PMC4967417

[14] J. L. Cataneo , H. Meidl , G. Joshi , et al., “The Association Between Body Mass Index and Lymph Node Harvest After Elective Colon Cancer Resections,” World Journal of Colorectal Surgery 11, no. 2 (2022): 21–26.

[15] Y. Fujieda , H. Maeda , K. Oba , et al., “Factors Influencing the Number of Retrieved Lymph Nodes After Colorectal Resection: A Retrospective Study From a Single Institute,” International Journal of Clinical and Experimental Pathology 11, no. 3 (2017): 1694–1700. PMC6958128 31938271

[16] Q. Liu , M. Huang , J. Yang , et al., “Association Between the Number of Retrieved Lymph Nodes and Demographic/Tumour‐Related Characteristics in Colorectal Cancer: A Systematic Review and Meta‐Analysis,” BMJ Open 13, no. 12 (2023): e072244. 10.1136/bmjopen-2023-072244 PMC10749009 38135324

[17] C. C. Foo , C. Ku , R. Wei , et al., “How Does Lymph Node Yield Affect Survival Outcomes of Stage I and II Colon Cancer?,” World Journal of Surgical Oncology 18, no. 1 (2020): 22. 31996214 10.1186/s12957-020-1802-6 PMC6990535

[18] R. O. Dillman , K. Aaron , F. S. Heinemann , and S. E. McClure , “Identification of 12 or More Lymph Nodes in Resected Colon Cancer Specimens as an Indicator of Quality Performance,” Cancer 115, no. 9 (2009): 1840–1848. 19208427 10.1002/cncr.24185

[19] S. Ichhpuniani , T. McKechnie , J. Lee , et al., “Lymph Node Harvest as a Predictor of Survival for Colon Cancer: A Systematic Review and Meta‐Analysis,” Surgery in Practice and Science 14 (2023): 100190. 39845856 10.1016/j.sipas.2023.100190 PMC11750021

[20] J. C. D. Paggio , Y. Peng , X. Wei , et al., “Population‐Based Study to Re‐Evaluate Optimal Lymph Node Yield in Colonic Cancer,” Journal of British Surgery 104, no. 8 (2017): 1087–1096. 10.1002/bjs.10540 PMC7938819 28542954

[21] C. H. A. Lee , S. Wilkins , K. Oliva , M. P. Staples , and P. J. McMurrick , “Role of Lymph Node Yield and Lymph Node Ratio in Predicting Outcomes in Non‐Metastatic Colorectal Cancer,” BJS Open 3, no. 1 (2018): 95–105. 30734020 10.1002/bjs5.96 PMC6354193

[22] J. Li , Y. z. Yang , P. Xu , and C. Zhang , “A Prognostic Model Based on the Log Odds Ratio of Positive Lymph Nodes Predicts Prognosis of Patients With Rectal Cancer,” Journal of Gastrointestinal Cancer 55, no. 3 (2024): 1111–1124. 38700666 10.1007/s12029-024-01046-2 PMC11347484

[23] X. Hu , L. Jiang , J. Wu , and W. Mao , “Prognostic Value of Log Odds of Positive Lymph Nodes, Lymph Node Ratio, and N Stage in Patients With Colorectal Signet Ring Cell Carcinoma: A Retrospective Cohort Study,” Frontiers in Surgery 9 (2023): 1019454. 36684239 10.3389/fsurg.2022.1019454 PMC9849566

[24] C. Fortea‐Sanchis , D. Martínez‐Ramos , and J. Escrig‐Sos , “The Lymph Node Status as a Prognostic Factor in Colon Cancer: Comparative Population Study of Classifications Using the Logarithm of the Ratio Between Metastatic and Non‐metastatic Nodes (LODDS) Versus the pN‐TNM Classification and Ganglion Ratio Systems,” BMC Cancer 18, no. 1 (2018): 1208. 30514228 10.1186/s12885-018-5048-4 PMC6280498

[25] J. D. Brierley , M. K. Gospodarowicz , and C. Wittekind , UICC TNM Classification of Malignant Tumours, 8th Edition (Willy Blackwell, 2017).

