# Supplementary material for: Perioperative and Oncological Outcomes of Colorectal Cancer Surgery in Obese Patients: A Multicenter Retrospective Study
Source: Ann Gastroenterol Surg. 2025 Dec 19;10(3):748–59. doi: 10.1002/ags3.70156 (PMC13178282; doi:10.1002/ags3.70156)
Supplement: Supplementary file 1 — Figure S1: Kaplan–Meier survival curves for relapse‐free survival (RFS) and overall survival (OS) according to BMI category in stage I–III colorectal cancer patients. Figure S2: ROC curve used to determine the optimal LODDS cut‐off for predicting recurrence. Figure S3: Recurrence‐free survival (RFS) and overall survival (OS) according to LODDS and adjuvant chemotherapy (ACT) status in stage III colorectal cancer, stratified by BMI category. Table S1: Association between BMI and Lymph Node Evaluation Parameters. Table S2: Association between LODDS and adjuvant chemotherapy according to BMI in patients with Stage III colorectal cancer. [file AGS3-10-748-s001.docx]

**Supporting information**

**
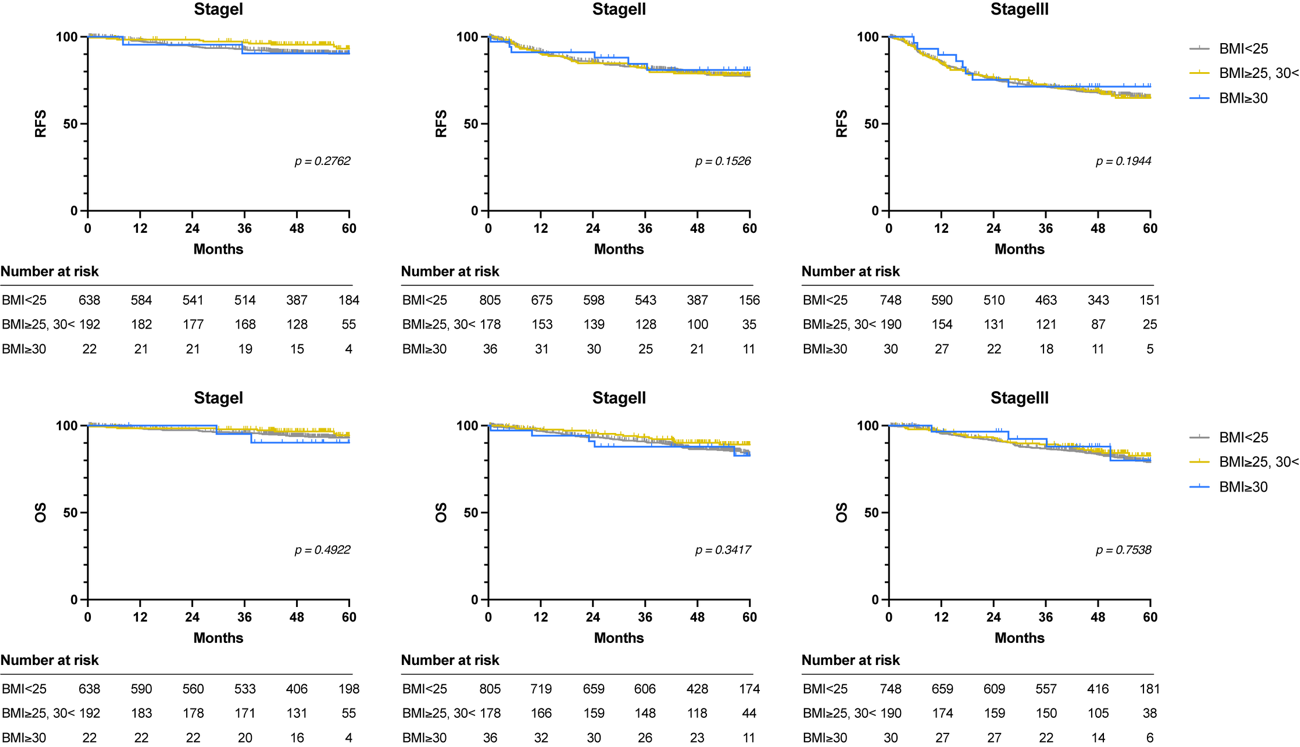
**

**Supplemental figure 1. Kaplan–Meier survival curves for recurrance-free survival (RFS) and overall survival (OS) according to BMI category in stage I–III colorectal cancer patients**

Patients were categorized into three BMI groups: BMI <25 (gray), BMI ≥25 and <30 (yellow), and BMI ≥30 (blue). RFS and OS curves are shown for each TNM stage: stage I (left panels), stage II (center panels), and stage III (right panels). The number at risk at each time point is shown below each panel. P values were calculated using the log-rank test.

Abbreviations: BMI, Body mass index; RFS, relapse-free survival; OS, overall survival.

**Supplemental figure 2. ROC curve used to determine the optimal LODDS cutoff for predicting recurrence.**

The ROC curve for recurrence is shown, with an area under the curve (AUC) of 0.6699. The optimal cutoff (LODDS = –0.7) was selected using the Youden index, corresponding to a sensitivity of 0.7467 and a 1-specificity of 0.4333. The diagonal line (yellow) represents the reference for a non-informative classifier.

**Supplemental figure 3. Recurrence-free survival (RFS) and overall survival (OS) according to LODDS and adjuvant chemotherapy (ACT) status in stage III colorectal cancer, stratified by BMI category.**

Kaplan–Meier curves of recurrence-free survival (RFS, top panels) and overall survival (OS, bottom panels) in patients with Stage III colorectal cancer stratified by BMI groups: BMI < 25 (left) and BMI ≥ 25 (right). Patients were further categorized based on LODDS values (< –0.7 vs. ≥ –0.7) and ACT status (ACT(–) and ACT(+)). Survival differences between LODDS < –0.7 and LODDS ≥ –0.7 in each ACT status were evaluated using the log-rank test. The number of patients at risk is displayed below each curve.

Abbreviations: BMI, Body mass index; LODDS, log odds of positive lymph nodes, ACT, adjuvant chemotherapy.

**Supplemental table 1.** Association between BMI and Lymph Node Evaluation Parameters

|  | **Non-Obesity**  **BMI <25**  **n = 2238** | **Mild-Obesity**  **BMI ≥25, <30**  **n = 576** | **Severe-Obesity**  **BMI ≥30**  **n = 91** | ***P value*** |
| --- | --- | --- | --- | --- |
| **# of examined LN**  <12  ≥12 | 478 (21.4%)  1760 (78.6%) | 157 (27.3%)  419 (72.7%) | 26 (28.6%)  65 (71.4%) | *0.0051* |
| **LODDS**  ≥−0.7  <−0.7 | 310 (13.8%)  1928 (86.2%) | 84 (14.6%)  492 (85.4%) | 22 (24.2%)  69 (75.8%) | *0.0366* |

BMI, body mass index; LN, lymph node; LODDS, log odds of positive lymph nodes

**Supplemental table 2.** Association between LODDS and adjuvant chemotherapy according to BMI in patients with Stage III colorectal cancer

|  | **LODDS <−0.7** | **LODDS ≥−0.7** | ***P value*** |
| --- | --- | --- | --- |
| **Stage III (n = 969)** | | | |
| **BMI <25 (n = 749)**  ACT (−)  ACT (+) | 170 (36.9%)  291 (63.1%) | 104 (36.1%)  184 (63.9%) | *0.8325* |
| **BMI ≥25 (n = 220)**  ACT (−)  ACT (+) | 49 (40.2%)  73 (59.8%) | 28 (28.6%)  70 (71.4%) | *0.0732* |

BMI, body mass index; LN, lymph node; LODDS, log odds of positive lymph nodes; ACT, adjuvant chemotherapy
